# Involvement of the Tubular ClC-Type Exchanger ClC-5 in Glomeruli of Human Proteinuric Nephropathies

**DOI:** 10.1371/journal.pone.0045605

**Published:** 2012-09-24

**Authors:** Monica Ceol, Emilia Tiralongo, Hans J. Baelde, Daniela Vianello, Giovanni Betto, Annunziata Marangelli, Luciana Bonfante, Marialuisa Valente, Mila Della Barbera, Angela D’Angelo, Franca Anglani, Dorella Del Prete

**Affiliations:** 1 Department of Medicine, Nephrology Unit and Kidney Histomorphology and Molecular Biology Laboratory, University of Padova, Padova, Italy; 2 Department of Pathology, Leiden University Medical Center, Leiden, The Netherlands; 3 S. Antonio Hospital, Division of Urology, Padova, Italy; 4 Department of Diagnostic Medical Science and Special Therapies, Institute of Pathological Anatomy, University of Padova, Padova, Italy; Fondazione IRCCS Ospedale Maggiore Policlinico & Fondazione D’Amico per la Ricerca sulle Malattie Renali, Italy

## Abstract

Glomerular protein handling mechanisms have received much attention in studies of nephrotic syndrome. Histopathological findings in renal biopsies from severely proteinuric patients support the likelihood of protein endocytosis by podocytes. ClC-5 is involved in the endocytosis of albumin in the proximal tubule.

**Aim:**

To investigate whether ClC-5 is expressed in the glomerular compartment and whether it has a role in proteinuric nephropathies. ClC-5 expression was studied using Real-time PCR in manually- and laser-microdissected biopsies from patients with type 2 diabetes (n 37) and IgA nephropathy (n 10); in biopsies of membranous glomerulopathy (MG) (n 14) immunohistochemistry for ClC-5 (with morphometric analysis) and for WT1 was done. Controls: cortical tissue (n 23) obtained from unaffected parts of tumor-related nephrectomy specimens.

**Results:**

ClC-5 was expressed at glomerular level in all biopsies. Glomerular ClC-5 levels were significantly higher in diabetic nephropaty and MG at both mRNA and protein level (p<0.002; p<0.01). ClC-5 and WT1 double-staining analysis in MG showed that ClC-5 was localized in the podocytes. ClC-5 ultrastructural immunolocalization was demonstrated in podocytes foot processes. Our study is the first to demonstrate that ClC-5 is expressed in human podocytes. The ClC-5 overexpression found in biopsies of proteinuric patients suggests that proteinuria may play a part in its expression and that podocytes are likely to have a key role in albumin handling in proteinuric states.

## Introduction

Glomerular protein handling mechanisms have received much attention in studies on nephrotic syndrome now that proteinuria has been recognized as an independent risk factor for both renal failure and cardiovascular disease. A considerable research effort has gone into understanding how the components of the slit diaphragm act as a barrier to macromolecular filtration, but little attention has been paid to whether resident glomerular cells, such as mesangial cells, podocytes and endothelial cells, have the potential to handle plasma proteins beyond slit diaphragm specialization [1]. Microalbuminuria is traditionally associated with a defect at glomerular level (loss of negative charge, slit diaphragm changes, overfiltration), but there is some evidence to suggest an important role for the tubules too [2].

ClC-5, belongs to the highly conserved ClC family of chloride channels and chloride/proton exchangers, and is part of the molecular complex involved at proximal tubular level in the endocytotic re-uptake of low-molecular-weight (LMW) proteins and of albumin too. This mechanism needs a very active receptor-mediated pathway, as Hryciw *et al.* neatly explained [3]. The endosomal H^+^/Cl^−^ exchanger ClC-5 has been located in the kidney using immunoabsorbed ClC-5-specific polyclonal antibodies raised against a synthetic peptide corresponding to the ClC-5 extracellular domain. It has been found in the proximal tubules, the thick ascending limb and the intercalated cells of the collecting ducts, but never in human glomeruli [4].

The endocytotic process at tubular level is well known, while little evidence is available on endocytosis in the glomerular compartment, although the first report of glomerular podocyte vacuolization in renal biopsies from severely proteinuric patients dates back to 1986, and pointed to the possibility of protein endocytosis by podocytes [5]. For podocytes to have a role in protein endocytosis, a sophisticated uptake system similar to the one in proximal tubular cells (PTC) would conceivably be required.

The aim of our study was to investigate whether ClC-5, as part of the macromolecular complex involved in albumin re-uptake, is expressed in the glomerular compartment, and whether it has a role in proteinuric nephropathies.

## Results

### ClC-5 Expression in the Glomerular Compartment of Human Biopsies

#### ClC-5 mRNA quantification

**Figure 1:**
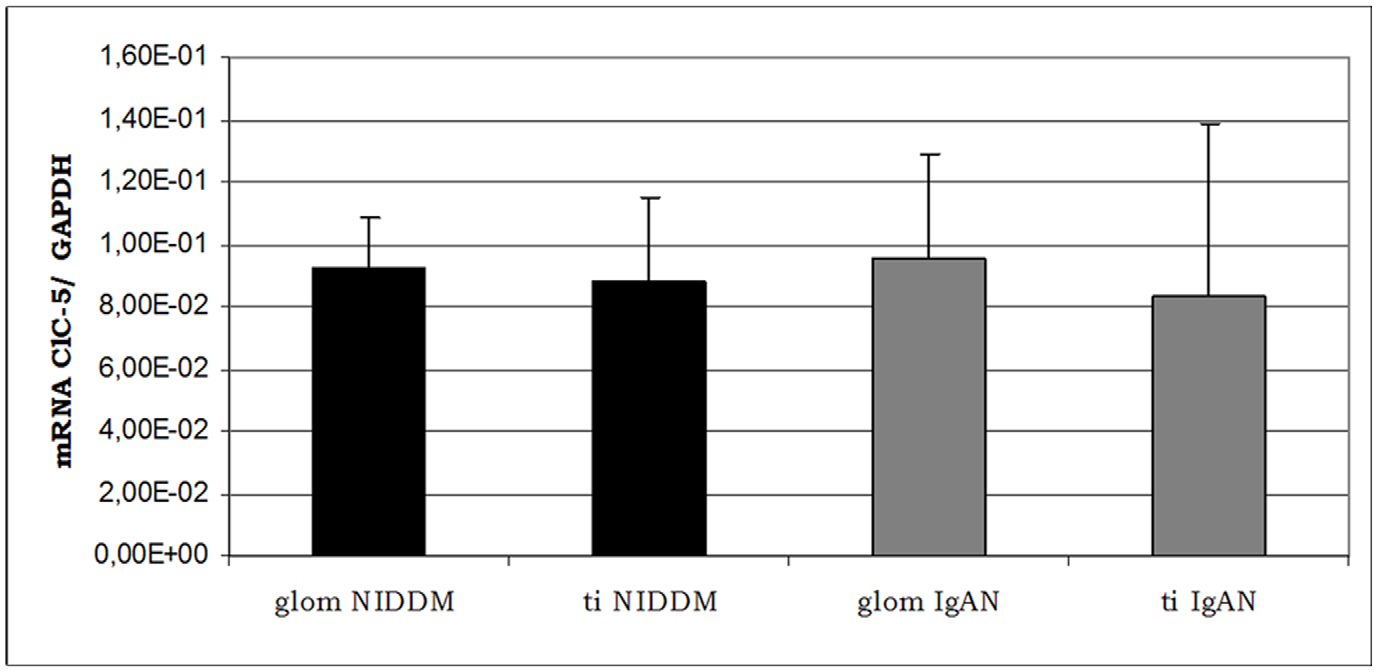
Real-Time quantification of ClC-5 in manually-microdissected biopsies of NIDDM and IgA patients. mRNA level of ClC-5, expressed as the ratio between the starting quantity means (SQm) of the target and housekeeping genes, in manually-microdissected biopsies from NIDDM and IgA patients at glomerular (glom) and tubulo-interstitial (ti) level.

Figure 1 shows ClC-5 mRNA levels, found by Real-Time PCR with SYBR Green dye, in the glomerular compartment of manually-microdissected biopsies from NIDDM (n 9) and IgAN (n 10) patients. No significant differences in ClC-5 expression were found between glomerular and tubulo-interstitial compartment in NIDDM and in IgAN.

**Figure 2:**
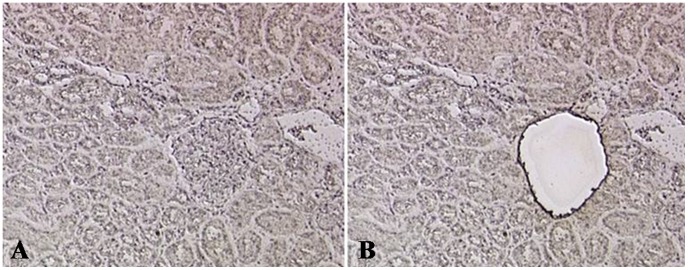
Histological picture of a laser microdissected biopsy. Example of laser-microdissection pre- (A) and post- (B) cut, showing that the glomerular tuft can be separated without any tubulo-interstitial contamination (Ematoxilin staining).

**Figure 3:**
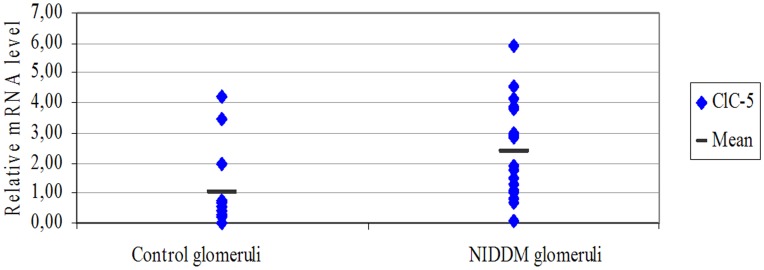
Real-Time quantification of ClC-5 in laser-microdissected biopsies of NIDDM and controls. mRNA level of ClC-5 in laser-microdissected glomeruli from NIDDM cases and controls. ClC-5 mRNA levels were significantly higher in NIDDM glomeruli than in control glomeruli (p<0.002). The relative expression levels of the gene of interest was obtained by dividing the gene’s expression level by the mean level of the different housekeeping genes. The relative levels of the controls were set to one.

To rule out the possibility of the glomerular compartment being contaminated by the proximal tubules and consequently falsifying our results, ClC-5 gene expression was analyzed on laser-microdissected glomeruli from other NIDDM cases (n 28) (Table 1, 2^nd^ group) and control patients (n 14). As shown in figure 2, laser-microdissection enabled us to obtain the glomerular tuft without any contamination from the tubulo-interstitial compartment. The study was conducted using a different technique, i.e. Real-Time PCR with TaqMan probes, and on different NIDDM patients with clinical pictures similar to those of the patients in group 1 (table 1). The analysis confirmed ClC-5 expression at glomerular level in both proteinuric and control biopsies. The mRNA level of ClC-5 was also significantly higher (p<0.002) in NIDDM patients than in controls (Fig. 3).

**Table 1:** Patients’ clinical details.

	NIDDM (1^st^ group)	NIDDM (2^nd^ group)	IgAN	MG
Duration of diabetes type II (years)	>5	>5	–	–
Mean age (years)	52	45	30	56
Gender	Male	Male	Male	10M/4F
Serum creatinine (mg/dl)	<1.8	<1.8	<1.8	<1.8
Urine protein (g/l)	1–1.5	1–1.5	1–1.5	2.5–3

Note: Conversion factors for units: serum creatinine in mg/dl to mol/L, x88.4.

#### ClC-5 protein quantification

Immunohistochemical analysis enabled us to ascertain the presence of ClC-5 protein in biopsies from controls and MG patients, at both glomerular and tubulo-interstitial level.

**Figure 4:**
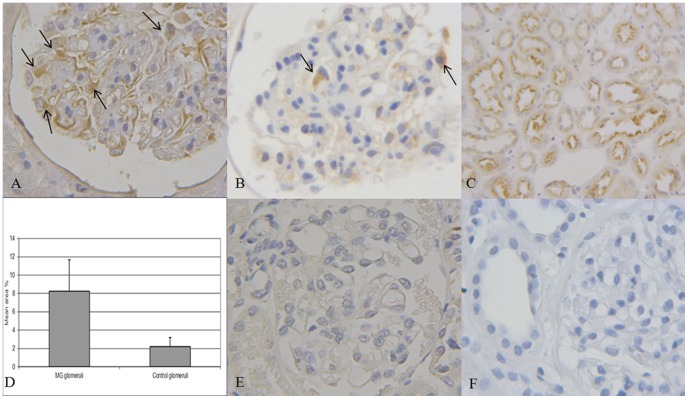
Immunoistochemical analysis in MG biopsies. A. Glomerular ClC-5 immunostaining in MG patient (400×). B. ClC-5 staining in a control glomerulus (400×). Black arrows indicate cytoplasmatic ClC-5 staining (400×). C. Proximal tubular cells used for internal control purposes showed ClC-5 apical and sub-apical staining (200×). D. ClC-5 quantification by morphometric analysis on MG and control glomeruli. ClC-5 signal was significantly higher in MG than in control samples (p<0.01). The quantity was expressed as the mean of the area covered by pixels, as a percentage. The average of the morphometric data in each group of biopsies is reported. E. Absence of glomerular ClC-5 staining in a patient with Dent’s disease (400×). F. Negative control obtained by incubating the sample with no primary antibody or with nonimmune rabbit IgG (400×).

In the glomerular compartment, ClC-5 staining was located in the cytoplasm of cells that looked like podocytes, judging from their morphology and localization (Fig. 4 A). The same staining pattern was observed in glomeruli of control kidneys (Fig. 4B). PTC used as an internal control showed ClC-5 apical and sub-apical staining (Fig. 4 C). On quantification by means of a morphometric analysis, ClC-5 deposition was significantly higher in MG glomeruli than in controls (0.084±0.03 vs 0.02±0.01; p<0.01) (Fig. 4 D). The immunohistochemistry performed in a biopsy of Dent’s disease patient allowed us to demonstrate the specificity of ClC-5 antibody: all the glomeruli and the corresponding tubule-interstitium were negative. A faint background was however visible in very few tubules probably caused by weak cross-reactivity of our ClC-5 antibody with the C-terminus of ClC-3 and ClC-4. In fact the antigen sequence shows around 66% overall sequence identity to both ClC-3 and ClC-4.

**Figure 5:**
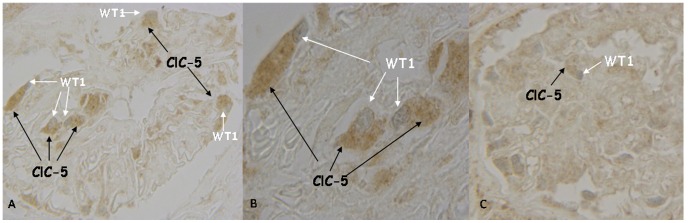
Double staining for ClC-5 and WT1 in MG and control biopsies. Co-localization of ClC-5 (cytoplasmatic brown stain) and WT1 (nuclear blu-grey stain) in control (C) and in MG (A) glomeruli to identify podocytes (400×). B. Oil image immersion of a detail of panel A (1000×).

To verify whether the cells expressing ClC-5 were podocytes, we performed double staining with ClC-5 and WT1 (a podocyte nuclear marker) in MG (Fig. 5 A–B) and control (Fig. 5 C) biopsies: WT1 staining was detected in the nuclei (as a blue-gray stain) of cells with cytoplasm staining for ClC-5 (brown stain).

Glomerular ClC-5 expression did not correlate (at mRNA or protein level) with proteinuria or serum creatinine in any of the proteinuric nephropathies.

**Figure 6:**
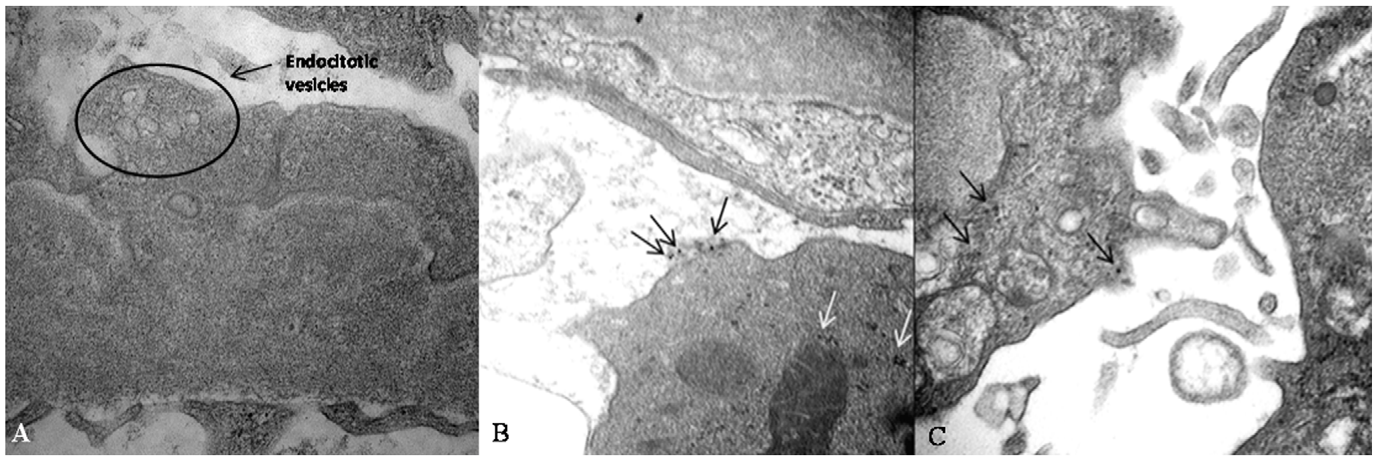
Immunolocalization of ClC-5 in ultrastucture of MG biopsies. A. Transmission electron microscopy (TEM) of MG biopsy revealed small vesicles in the secondary foot processes of podocytes (direct magnification 9000×). B–C. Ultrastructural immunolocalization of ClC-5 in MG biopsy. Arrows indicate some of the gold particles located in podocytes (direct magnification 40000×).

The ClC-5 protein level was higher in MG biopsies with stage II, as defined by TEM analysis, than in the other stages, though the difference was not statistically significant (data not shown). There were also intracellular vesicular structures in the secondary foot process of podocytes on ultrastructure of our MG biopsies (Fig. 6 A). Ultrastructural immunolocalization of ClC-5 was demonstrated in podocytes (Fig. 6 B–C).

## Discussion

Our findings are the first to reveal ClC-5 expression in human glomeruli at both mRNA and protein level. While the presence and role of ClC-5 in the tubular compartment is well known [4], to the best of our knowledge there have been no previous reports of ClC-5 expression in human glomeruli.

Ours is also the first demonstration that ClC-5 is overexpressed in the glomeruli of proteinuric patients, pointing to the presence of an endocytotic machinery similar to that of PTC.

Finding ClC-5 gene expression in the glomerular compartment of manually microdissected glomeruli prompted us to check for any proximal tubule contamination. To do so, we repeated the gene expression experiments on laser-microdissected glomeruli and conducted an immunohistochemical analysis, which confirmed both the presence of ClC-5 in the glomeruli and its overexpression in proteinuric nephropathies.

The reliability of our findings is confirmed by the reproducibility of our results, obtained in different proteinuric diseases and using different techniques (Real-Time PCR with SYBR Green, Real-Time PCR with TaqMan probes, and immunohistochemistry).

Albumin endocytosis by the proximal tubuli is a well-known phenomenon [2], but little information is available on the mechanisms governing endocytosis in the glomerular compartment. Evidence of protein endocytosis by the podocytes derives from the clinical assessment of renal biopsies from severely proteinuric patients, which commonly show signs of extensive protein vacuolation by podocytes [5]. Tracer experiments using ferritin have also identified this large protein in podocyte lysosomes, suggesting that podocytes have an endocytic recovery function for ferritin that passes through the glomerular basement membrane [6]. More recently, Eyre *et al.* [7] demonstrated the accumulation of endocytosed albumin in the intracellular vesicles of podocytes, both in vitro and in vivo, using fluorescence confocal microscopy and electron microscopy. Koop *et al.* [8] also found protein droplets in the podocyte cell bodies and major processes from Dahl salt-sensitive rats. In puromycin aminonucleoside nephrotic (PAN) rats, Tojo et al [9] recently demonstrated that albumin is filtered via other pathways beyond the slit diaphragm, which include endocytosis by endothelial cells, uptake by podocytes and entrapment in the paramesangium. Again in PAN rats, using the immunogold method for albumin, Kinugasa et al [10] found many particles on the podocytes’ surface. Intracellular vesicular structures in the secondary foot process of podocytes was also observed with transmission electron microscopy (TEM) on our MG biopsies and ultrastructural immunolocalization confirmed the presence of ClC-5 in podocytes (Fig.6). Although all the above-mentioned reports confirm that podocytes can endocytose proteins, the mechanisms behind this endocytotic process at glomerular level remains obscure. It is also still not clear whether this is linked exclusively to proteinuric disease or occurs in healthy people too.

Finding ClC-5 expressed in normal glomeruli as well would seem to indirectly confirm the latter hypothesis, suggesting a role for endocytosis in both normal and pathological glomeruli. Identifying podocytes as the main cells involved in ClC-5 expression would also suggested that this epithelial cell plays a key part in the protein’s endocytotic re-uptake.

At proximal tubular level ClC-5 and Megalin are part of the molecular complex involved in the endocytotic re-uptake of both LMW proteins and albumin. The role of this macromolecular complex at tubular level is well known, while available information on Megalin at glomerular level only comes from animal models. The discovery of Megalin as a pathogenic antigen of Heymann Nephritis in rats [11] dates back to 1982, but it is only recently that Megalin was found expressed in human podocytes of Fabry Disease patients [12], meaning that Megalin is probably to be found in human glomeruli too, and may act as a pathogenic antigen.

It has recently been demonstrated that mouse podocytes use FcRn, an IgG and albumin transport receptor, to clear proteins that would otherwise clog the slit diaphragm [13]. In polarized epithelia, the FcRn receptor has been shown to traffic IgG from the apical and basolateral membrane into the recycling endosomes via a transcytotic mechanism that is still poorly understood. The FcRn receptor has also been identified in the podocytes of adult human glomeruli [14]–[15]. Which binding mechanism is used by ClC-5 at glomerular level remains to be discovered.

Finding that ClC-5 is overexpressed in the glomeruli of patients with proteinuric diseases, prompted us to hypothesize a role for this protein in the pathophysiology of proteinuria, probably relating to an effect that counteracts the larger quantity of filtered proteins. It is hard to say whether a healthy cell function can adapts to a pathological environment, or whether such an adaptive process may become maladaptive in disease. The reason why no correlation emerged between glomerular ClC-5 expression, at mRNA or protein level, and proteinuria in all the proteinuric nephropathies we studied (NIDDM, IgAN, MG) might lie in the fact that not all our patients underwent ACE inhibitor therapy wash-out, and in the small number of patients considered in this study.

In conclusion, our data indicate that ClC-5 in glomeruli, and at podocyte level in particular, may have a role in protein endocytosis. This could be another route for glomerular albumin clearance in proteinuric nephropathy.

## Materials and Methods

### Ethics Statement

All clinical investigations were conducted according to the principles expressed in the Declaration of Helsinki and patients’ data were analyzed anonymously. All patients gave their informed, written consent. The study was approved by Ethics Committee for experimental studies of Azienda Ospedaliera of Padova protocol number 0027778 (29/5/2012).

### Biopsies

Three different groups of human renal biopsies were analyzed for this study: the first included manually-microdissected glomeruli from cortical biopsies of 10 patients with a diagnosis of IgA nephropathy (IgAN) and 9 patients with diabetic nephropathy (NIDDM); the second group contained laser-microdissected glomeruli from biopsies of 28 NIDDM patients and 14 controls, obtained from sites remote from the tumor-bearing renal tissue; the third group consisted of 14 biopsies from patients with a diagnosis of membranous glomerulopathy (MG) assessed by optical, immunofluorescence and electron microscopy, plus 6 control cortical tissues disclosing a normal morphology and a negative immunofluorescence. Moreover in order to assess the specificity of ClC-5 antibody we performed immunohistochemistry in renal biopsy of a patient with Dent’s disease who carries the stop codon mutation R34X in the ClC-5 protein. This mutation leads to a truncated protein that completely lacks the carboxy terminal domain.

Renal biopsies were obtained under ultrasound guidance using a 14-gauge needle. Only the biopsies providing a sample sufficient for both standard pathological examination and molecular biology analyses were considered in this study.

The patients’ main clinical characteristics of interest for the purposes of this study are given in Table 1.

All patients gave their informed, written consent.

### ClC-5 Gene Expression Study in Manually-microdissected Biopsies

ClC-5 expression was analyzed using Real-Time PCR with SYBR Green 1.

Immediately after obtaining the biopsy, approximately one tenth of each specimen was kept in physiological solution containing 100 U of RNase inhibitor (Perkin Elmer, USA) on ice. Glomeruli were isolated under a stereo microscope (Zeiss, Germany) and the corresponding tubulo-interstitium was also collected. Total RNA extraction and Reverse Transcription (RT) were performed as described elsewhere [16].

#### Real-Time PCR quantification using SYBR Green I

Real-Time PCR with SYBR Green dye was used to quantify ClC-5 expression, using glyceraldehyde phosphate dehydrogenase (GAPDH) as the housekeeping gene, with the iCycler Thermal Cycler (BioRad, Hercules, CA, USA). The PCR standards for ClC-5 and GAPDH were obtained as described elsewhere [16].

The primer sequences were: ClC-5 Fw 5′-CAGAGTGGAATAGTTGGTC-3′, Rw 5′-AGAGATACGGCAAGGAAG-3′; GAPDH Fw 5′-GAAGGTGAAGGTCGGAGT-3′, Rw 5′-TGGCAACAATATCCACTTTACCA-3′. The size of the PCR products amplified with primers for SYBR Green I analysis were: GAPDH 92 bp; CLC-5 121 bp. Real-time PCR quantification was done starting from the same quantity of cDNA (2 µl ).

The optimal concentration of primers (300 nmol/l) and MgCl_2_ (3 mmol/l) was established in preliminary experiments. The thermal cycling profile for GAPDH consisted of: step 1, 95°C for 5 minutes; step 2, 94°C for 30 seconds; step 3, 60°C for 30 seconds (steps 2 and 3 were repeated for 40 cycles); and step 4, melting curve. For ClC-5, the profile consisted of: step 1, 95°C for 5 minutes; step 2, 94°C for 45 seconds; step 3, 54°C for 45 seconds (repeating steps 2 and 3 for 40 cycles); step 4, melting curve. As SYBR Green I also binds to primer dimers forming nonspecifically during all PCR reactions, the most favorable temperature for analyzing the specific product had to be established. Melting curve analysis was used to confirm the specificity of the amplification products, as follows: step 1 at 50°C for 15 seconds; step 2 increasing the temperature by 0.5°C every 15 seconds from 50°C up to a final temperature of 95°C. The quantification data were analyzed with iCycler software and expressed as the ratio between the starting quantity means (SQm) of the target and housekeeping genes.

### ClC-5 Gene Expression Study in Laser-microdissected Glomeruli

The samples from the second group of patients (NIDDM and controls) underwent laser microdissection, RNA extraction, RT and Real-Time PCR analysis with Taqman probes on isolated decapsulated glomeruli, using three different housekeeping genes - GAPDH, hypoxanthine-phosphoribosyl-transferase (HPRT), and TATA box binding protein (TBP). The primer sequences were: HPRT Fw: TGACACTGGCAAAACAATGCA, Rw: GGTCCTTTTCACCAGCAAGCT; TBP Fw: CACGAACCACGGCACTGATT, Rw: TTTTCTTGCTGCCAGTCTGGAC. Quantification of ClC-5 was performed as described elsewhere [17]. CT values of PCR for ClC-5 varied between 33–40, while those for the housekeeping genes were between 27–29.

Expression studies were performed in manually- and laser-microdissected glomeruli using the same primer sequences for GAPDH and ClC-5.

### Immunohistochemical Analyses

#### ClC-5 staining

ClC-5 staining was investigated on 14 kidney biopsies of patients with MG and 6 cortical tissues obtained from unaffected parts of tumor-related nephrectomy specimens. The immunohistochemical analysis was performed on formalin-fixed, paraffin-embedded sections using an indirect immunoperoxidase method with a primary rabbit anti-human ClC-5 polyclonal antibody directed against the cytoplasmic carboxy terminal domain (SIGMA Aldrich, St. Louis, USA). After deparaffinization and rehydration in an ethanol series, sections were treated with 3% H_2_O_2_ in 50 mM phosphate-buffered saline (PBS) (pH 7.4) for 30 min at room temperature to remove the endogenous peroxidase. Antigens were retrieved by heating in a microwave oven (500 W for 5 min×3 times in 5 mM citrate buffer solution pH 6.0). After cooling, sections were rinsed in PBS and incubated in blocking solution containing 2% normal goat serum for 30 min in a humidified chamber before incubation overnight at 4°C with primary Abs (1∶100 dilution). Sections were then rinsed and treated with DakoCytomation EnVision + System-HRP Labeled Polymer anti-rabbit (DAKO, Glostrup, Denmark) in a humidified chamber at room temperature for 30 min. The signal was visualized by 3,3′-diaminobenzidine-tetrachloride (DAB, DAKO Corporation, Carpinteria, CA, USA) and sections were counterstained with hematoxylin and analyzed under the light microscope. The specificity of the immunolabeling was confirmed by incubation without primary or secondary antibody or with nonimmune rabbit IgG (SIGMA Aldrich, St Louis USA).

#### Morphometric analysis

The immunohistochemical signal of ClC-5 was quantified by morphometric analysis (using Image Pro-Plus software, Media Cybernetics) at 200× magnification. The signal was acquired for all fields with the same characteristic of brightness and contrast selecting all glomeruli from each biopsy (a total of 89 glomeruli for MG and 32 glomeruli for controls) and expressing the quantity as the mean area covered by pixels, as a percentage.

#### WT1 and ClC-5 double staining

A WT1 and ClC-5 double staining was performed on kidney biopsies of MG patients to establish which cells were involved in ClC-5 expression at glomerular level.

Paraffin-embedded tissues were cut into 4 µm sections. After deparaffinization, endogenous peroxidase was inhibited by incubation with 3% H_2_O_2_ in 50 mM PBS (pH 7.4) for 30 min. The specimens were rinsed in PBS and antigens were retrieved by heating in a microwave oven (500 W for 5 min×3 times in citrate buffer). After cooling, the sections were rinsed again in PBS, blocked in 2% normal goat serum for 30 min, and incubated with rabbit anti-human ClC-5 overnight at 4°C. The specimens were rinsed in PBS, reacted with DakoCytomation EnVision + System-HRP Labeled Polymer anti-rabbit in a humidified chamber for 30 min and stained with DAB (brown stain). Then they were incubated again in 3% H_2_O_2_ in 50 mM PBS for 30 min and rinsed in PBS. After blocking with 2% normal goat serum solution for 30 min, they were incubated overnight at 4°C with rabbit anti-human WT1 (1∶100) polyclonal antibody (Santa Cruz Biotechnology Inc, CA, USA). The specimens were rinsed in PBS and treated with DakoCytomation EnVision + System-HRP Labeled Polymer anti-rabbit (DAKO, Glostrup, Denmark) in a humidified chamber for 30 min. The WT1 signal (blue-gray stain) was visualized with the Vector SG substrate kit for peroxidase (VECTOR Laboratories, Burlingame, USA). No hematoxylin counterstaining was performed.

### Transmission Electron Microscopy (TEM)

TEM was performed on all MG biopsies. Semithin sections (0.5 µm or less) were evaluated at light microscope first, before proceeding to consider ultrathin sections. The sections were stained with uranyl acetate and lead citrate and examined under a Hitachi H-7000 electron microscope equipped with digital camera.

### Immunogold Labeling

For ultrastructural analysis, renal biopsies were fixed by immersion in modified Karnovsky’s solution (2% paraformaldehyde/0.5%glutaraldehyde) in 0.1 M sodium phosfate buffer, pH 7.2, dehydrated in crescent ethanol concentration and embedded in LRWhite medium (EMS, Hatfield, PA, USA). Biopsies ultrathin sections, picked up on nikel grids, underwent to a post-embedding immunogold labeling by using 1∶100 rabbit anti-CLCN5 antibody (Sigma-Aldrich, Saint Louis, MO, USA) in TBS-1%BSA buffer at 4°C overnight followed by 1 h of incubation with (1∶40) goat anti-rabbit IgG 15-nm gold particles conjugated (BBInternational, Cardiff, UK). Control sections were incubated with secondary antibodies alone. After staining with 1% uranyl acetate in aqueous solution for five minutes, grids with sections were observed on Hitachi H-7000 (Hitachi, Japan) transmission electron microscope.

### Statistical Analysis

Student’s t-test and regression analysis were performed. A p-value ≤0.05 was considered statistically significant.
